# Lipoproteins LDL versus HDL as nanocarriers to target either cancer cells or macrophages

**DOI:** 10.1172/jci.insight.140280

**Published:** 2020-12-17

**Authors:** Tarik Hadi, Christophe Ramseyer, Thomas Gautier, Pierre-Simon Bellaye, Tatiana Lopez, Antonin Schmitt, Sarah Foley, Semen Yesylevskyy, Thibault Minervini, Romain Douhard, Lucile Dondaine, Lil Proukhnitzky, Samir Messaoudi, Maeva Wendremaire, Mathieu Moreau, Fabrice Neiers, Bertrand Collin, Franck Denat, Laurent Lagrost, Carmen Garrido, Frederic Lirussi

**Affiliations:** 1 INSERM, U1231, Label LipSTIC, and Ligue Nationale contre le Cancer, Dijon, France.; 2 Université de Bourgogne-Franche Comté, France.; 3 NYU Langone Medical Center, department of Cardiac Surgery, New York, New York, USA.; 4 Laboratoire Chrono Environnement UMR CNRS 6249, Université de Bourgogne Franche-Comté, Besançon Cedex, France.; 5 Anti-cancer Center George-François Leclerc, CGFL, Dijon, France.; 6 Department of Physics of Biological Systems, Institute of Physics of the National Academy of Sciences of Ukraine, Kyiv, Ukraine.; 7 BioCIS, Univ. Paris-Sud, CNRS, Univ. Paris-Saclay, Châtenay-Malabry, France.; 8 ICMUB, Institut de Chimie Moléculaire de l’Université de Bourgogne, UMR6302, CNRS, Université Bourgogne Franche-Comté, Dijon, France.; 9 Centre des Sciences du Goût et de l’Alimentation, INRA, CNRS, Bourgogne Franche-Comté University, France.; 10 University Hospital of Besançon (CHU), France.

**Keywords:** Oncology, Therapeutics, Cancer immunotherapy, Colorectal cancer, Lipoproteins

## Abstract

In this work, we have explored natural unmodified low- and high-density lipoproteins (LDL and HDL, respectively) as selective delivery vectors in colorectal cancer therapy. We show in vitro in cultured cells and in vivo (NanoSPECT/CT) in the CT-26 mice colorectal cancer model that LDLs are mainly taken up by cancer cells, while HDLs are preferentially taken up by macrophages. We loaded LDLs with cisplatin and HDLs with the heat shock protein-70 inhibitor AC1LINNC, turning them into a pair of “Trojan horses” delivering drugs selectively to their target cells as demonstrated in vitro in human colorectal cancer cells and macrophages, and in vivo. Coupling of the drugs to lipoproteins and stability was assessed by mass spectometry and raman spectrometry analysis. Cisplatin vectorized in LDLs led to better tumor growth suppression with strongly reduced adverse effects such as renal or liver toxicity. AC1LINNC vectorized into HDLs induced a strong oxidative burst in macrophages and innate anticancer immune response. Cumulative antitumor effect was observed for both drug-loaded lipoproteins. Altogether, our data show that lipoproteins from patient blood can be used as natural nanocarriers allowing cell-specific targeting, paving the way toward more efficient, safer, and personalized use of chemotherapeutic and immunotherapeutic drugs in cancer.

## Introduction

Among promising immunotherapeutic approaches in cancer are those targeting macrophages. Macrophages are present in substantial amounts in most solid tumors and influence tumor growth or regression through inflammatory and metabolic switch (1, 2). Macrophages, depending on their inflammatory status, can have a phenotype either tolerogenic (protumoral) or cytotoxic (antitumoral). Those that infiltrate the tumor are tolerogenic. The process of switching the polarization of those macrophages so they become cytotoxic is being investigated by many scientists (3). We recently demonstrated that the stress-inducible heat shock protein-70 (HSP70) was abundantly secreted by tumor cells and favored protumor phenotype in macrophages. Accordingly, depletion of HSP70 induced tumor regression via a massive intratumor recruitment of cytotoxic macrophages (4).

Concerning cancer chemotherapy, platinum-derived drugs such as cisplatin are major compounds in cancer treatment, particularly in colorectal cancer. However, besides high efficiency, cisplatin cytotoxicity causes cellular damage in healthy tissues, leading to adverse side effects such as renal and liver failure (5), pulmonary fibrosis, or increased cardiovascular events (6), dramatically limiting the dose of cisplatin that can be used on patients, thereby limiting its efficacy.

More specific cell targeting, toxicity, and solubilization issues have prompted the development of nanovectorization approaches. Hence, cisplatin encapsulation into liposomes is considered a promising strategy to reduce the amount of free cisplatin in the plasma and to specifically deliver it to target tissues. While most of these attempts did not show significant toxicity reduction, a few studies successfully passed phase II clinical trials but still need phase III validation (7). Another approach to improve cisplatin efficacy in colon cancer treatment has been recently proposed through the creation of orally administered squalenoylated nanoparticles loaded with cisplatin (8).

In contrast to these artificial lipid nanovectors, in this work, we have explored natural lipoproteins (LDL and HDL) as nanocarriers for colorectal cancer drugs. We studied cisplatin and the potential immunotherapeutic HSP70-targeting drug (AC1LINNC) binding to purified native human lipoproteins, as well as the influence of such complexation on their selective transport to colorectal cancer cells or macrophages.

## Results

### Pharmacodynamic parameters of LDL and HDL.

**Figure 1:**
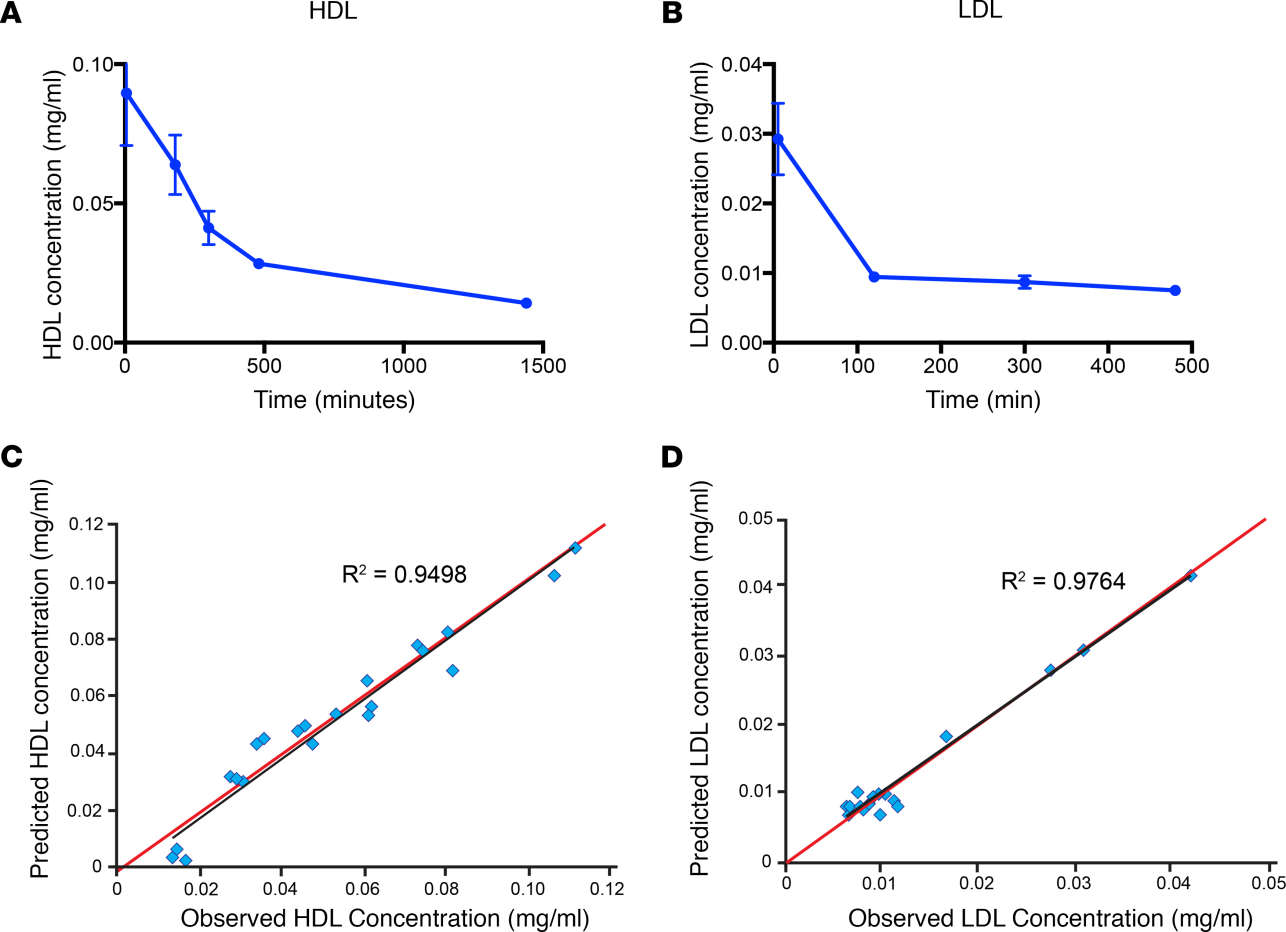
Pharmacokinetics parameters of LDL and HDL in mice. (**A** and **B**) Balb/c mice were injected i.v. with LDL-Bodipy (**A**) or HDL-Bodipy (**B**), 100 μL lipoprotein (1 mM cholesterol), 5 mice per group. A total of 100 μL of blood sample was dragged at the indicated times. Lipoproteins were extracted by ultracentrifugation, and Bodipy-bound HDL/LDL concentration was assessed by fluorimetry. Values are represented as mean ± SEM. Mean PK parameters for each condition were compared in order to sort out any differences. (**C** and **D**) In addition to NCA, a population PK approach was used. This approach allows, with a limited number of samples per animal, to determine typical and individual compartmental PK parameters (ka, Cl/F, distribution volume of central compartment, intercompartmental clearances, and distribution volume of peripheral compartments) and the interindividual variabilities associated to those PK parameters. Data are presented as correlation between the predicted and observed concentrations of HDL (**C**) and LDL (**D**) using this approach. ka, constant of absorption rate; Cl, clearance; F, bioavailability.

We first determined the pharmacodynamic (PK) parameters of lipoproteins in Balb/c mice to choose the optimal time for tissue distribution assessment. Purified HDLs and LDLs were labeled with DOTA-Bodipy-NCS and injected i.v. into mice. Mean concentration time courses are presented in Figure 1, A and B. PK parameters were relatively consistent between animals for HDL (Figure 1A and Supplemental Table 1; supplemental material available online with this article; https://doi.org/10.1172/jci.insight.140280DS1). Parameters were more variable between animals for LDL (Figure 1B and Supplemental Table 2), due to a rapid decrease in labeled LDL concentration during the first minutes following i.v. injection. C_max_ was rapidly reached after HDL i.v. injection and was about 0.09 mg/mL for a dose of 0.615 mg. Mean exposure between the first and the last measurements (i.e., AUClast) was equal to 45.79 mg/mL per minute and represented about 80% of the total exposure. Clearance was low, leading to a median terminal half-life of 14 hours. After 300 minutes, LDL concentrations were not always quantifiable. Mean exposure between the first and the last measurements (i.e., AUClast) was equal to 4.73 mg/mL per minute. Clearance was higher than for HDL, with a median terminal half-life of about 10 hours.

The goodness-of-fit plots (Figure 1, C and D) show a good correlation between observed and predicted concentrations, assuming that HDL and LDL followed a bicompartmental model. The major variability was linked to the volume of distribution in the central compartment (i.e., blood) (Supplemental Table 3). The developed model enabled us to determine the optimal limited sampling strategy for our further experiments.

### Tissue distribution and cancer cell specificity of LDL versus HDL in tumor-bearing mice.

**Figure 2:**
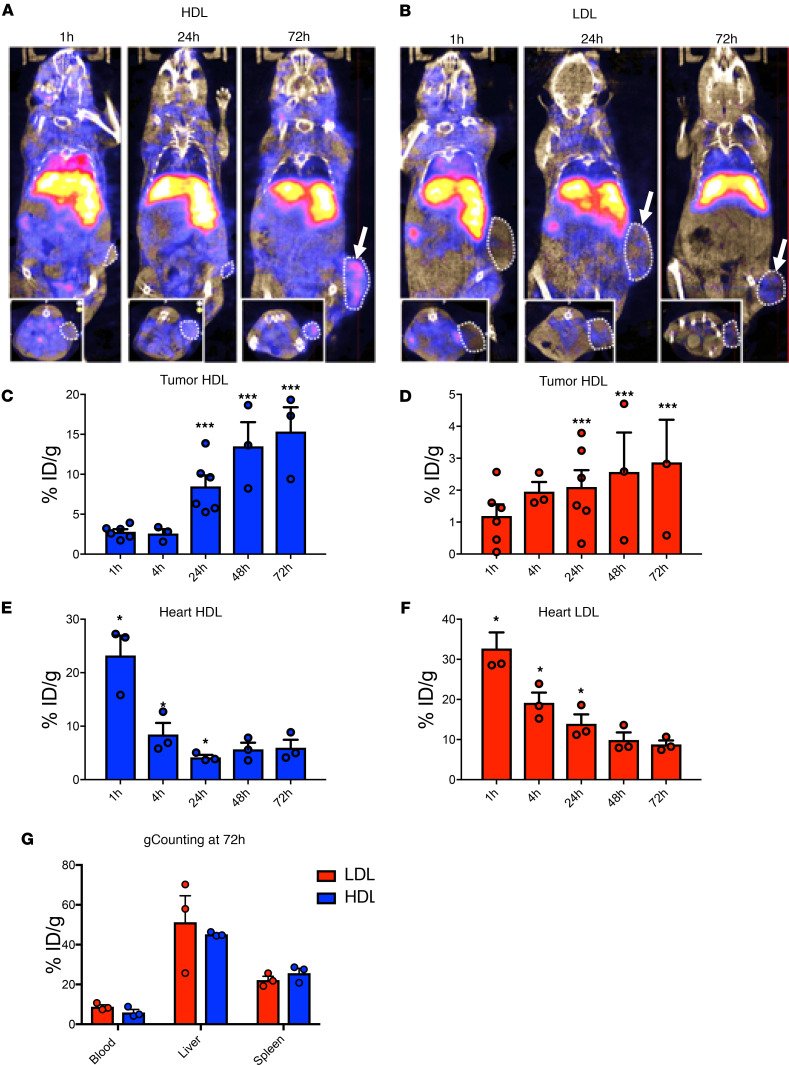
LDL- and HDL-Bodipy biodistribution in tumor-bearing mice. (**A** and **B**) Female Balb/c mice (*n* = 3) were grafted by s.c. injection of 1 × 10^6^ CT-26 colorectal tumor cells. Insets show transverse images. When tumor reached approximately 300 mm^3^, tumor-bearing mice were given 5 μg ^111^In-DOTAGA-HDL (**A**) or ^111^In-DOTAGA-LDL (8−10 MBq) (**B**) by i.v. injection. SPECT/CT dual imaging was performed 1, 24, and 72 hours after the injection of the radiolabeled conjugate using a NanoSPECT/CT small animal imaging tomographic γ-camera. CT (55 kVp, 34 mAs) and helical SPECT acquisitions were performed in immediate sequence. Radioactivity was measured with a scintillation γ-counter from tumor and heart. Data were then converted to percentage of injected dose per gram of tissue (%ID/g). SPECT/CT fusion image was obtained using the InVivoScope software. (**C**–**G**) Radioactivity in tumors (**C** and **D**, *n* = 6), heart (**E** and **F**, and *n* = 3), and blood, liver, and spleen (**G** and *n* = 3) was measured was measured with a scintillation γ-counter. Data are presented as mean value ± SEM. *P < 0.05, ***P < 0.001. *P* values were calculated using 1-way ANOVA.

For in vivo tissue distribution and cellular uptake of LDL and HDL, lipoproteins were labeled with radiolabeled ^111^Indium (^111^In) DOTA-Bodipy-NCS and injected i.v. into colorectal CT-26 tumor–bearing mice. This was reached 12 days after s.c. injection of CT-26 cancer cells into the left flank of Balb/c mice. Labeled LDL and HDL in vivo distribution was followed by single-photon emission computed tomography (SPECT-CT) and fluorescence microscopy. Lipoprotein uptake in the tumor and different organs (liver, spleen, heart, kidneys, bladder, and blood) was visualized over 72 hours in the whole animal (Figure 2, A and B). As expected, the highest levels of both LDLs and HDLs were found in the liver (Figure 2G and Supplemental Figure 1A). However, 24 hours after systemic injections, lipoproteins could be visualized in the tumors, reaching a peak between 48 and 72 hours (Figure 2, A and B). HDL and LDL increase in tumors inversely correlated with their decrease in the heart (Figure 2, C–F), indicative of lipoprotein decrease in the bloodstream. Of note, low amounts of both HDL and LDL were observed in the bladder throughout the experiment (Supplemental Figure 1B), suggesting that both lipoproteins were not rapidly cleared in the urine. It is worth noting that LDL was probably more difficult to visualize within the tumor because of its much faster clearance (Figure 1B).

**Figure 3:**
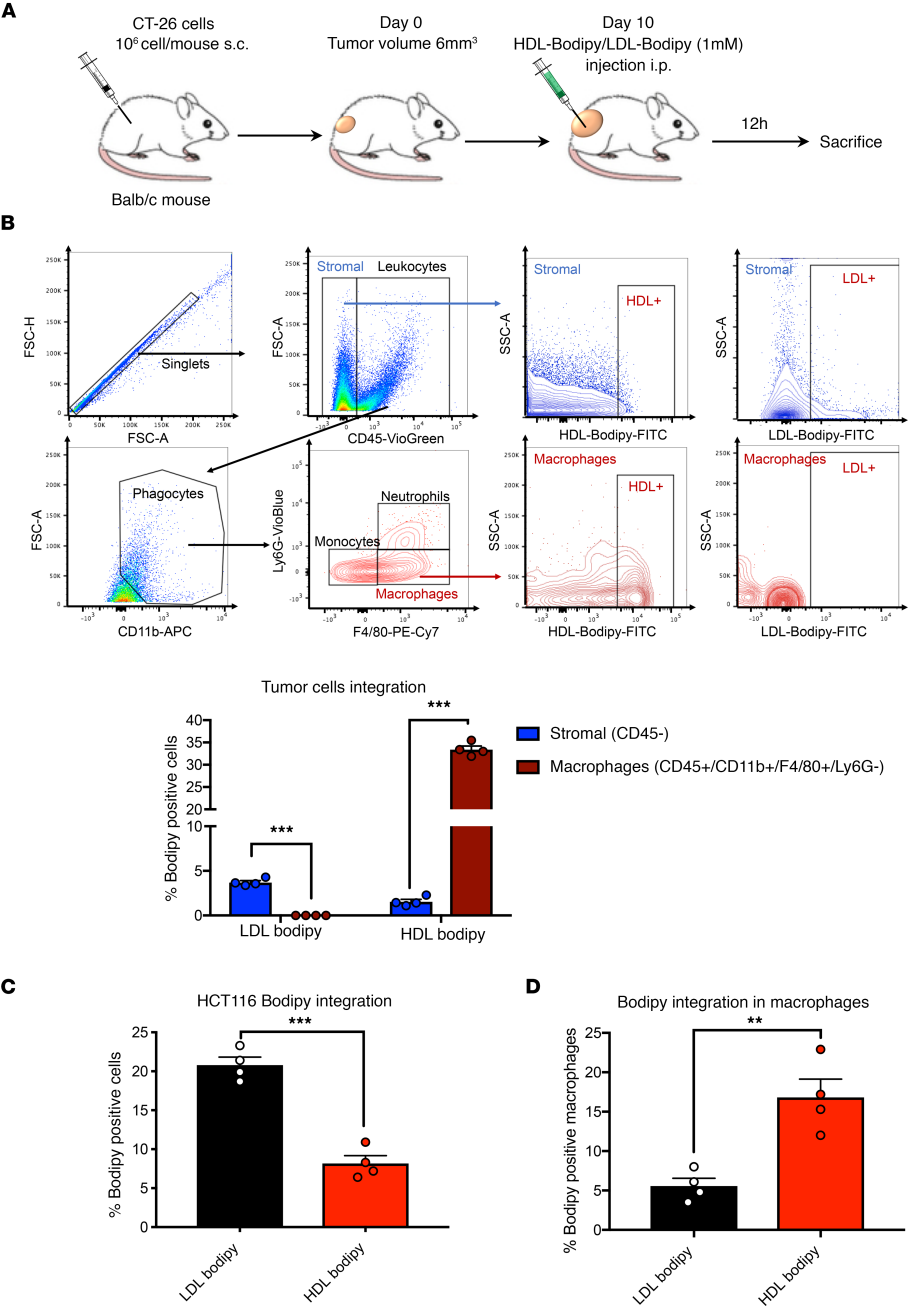
LDL-Bodipy accumulates in cancer cells, while HLD-Bodipy preferentially target macrophages. (**A**) Tumor cells and macrophages were isolated by FACS from dissociated CT-26 tumors of mice that have received LDL- or HLD-Bodipy (100 μM cholesterol) 12 hours before. (**B**) Representative gating strategy by flow cytometry for macrophages (CD45^+^/CD11b^+^/F4/80^+^/Ly6G^–^) and stromal cells (CD45^–^). Percentages of Bodipy^+^ cancer cells (F4/80^–^, blue bars) and macrophages (F4/80^+^, red bars) are represented as mean value ± SEM. *n* = 4, ***P < 0.001. (**C** and **D**) Human colorectal HCT116 cancer cells (**C**) and human primary macrophages (**D**) were treated for 6 hours with LDL-Bodipy or HDL-Bodipy (100 μM cholesterol). Percentages of Bodipy^+^ cells are represented as mean values. *n* = 4, **P < 0.01, ***P < 0.001. *P* values were calculated using 2-tailed unpaired *t* tests.

We next aimed to determine LDL and HDL subpopulation fates within the tumor. Twelve hours after injection of Bodipy-bound HDL and LDL, the tumors were dissociated and submitted to FACS analysis (Figure 3, A and B). Interestingly, we observed that LDL-Bodipy was preferentially up taken by CD45^–^ cells, which are predominantly tumor cells, while HDL-Bodipy preferentially accumulated in macrophages (CD45^+^CD11b^+^F4/80^+^Ly6G^–^, Figure 3B). This differential targeting of LDL versus HDL was confirmed in vitro using human cultured cells. Indeed, colorectal cancer HCT116 cells preferentially accumulated LDL-Bodipy (Figure 3C) while macrophages (from healthy volunteer buffy coats) mainly took up HDL-Bodipy (Figure 3D).

### HDL as vectors for potential immunotherapeutic agents such as the HSP70-targeting AC1LINNC.

**Figure 4:**
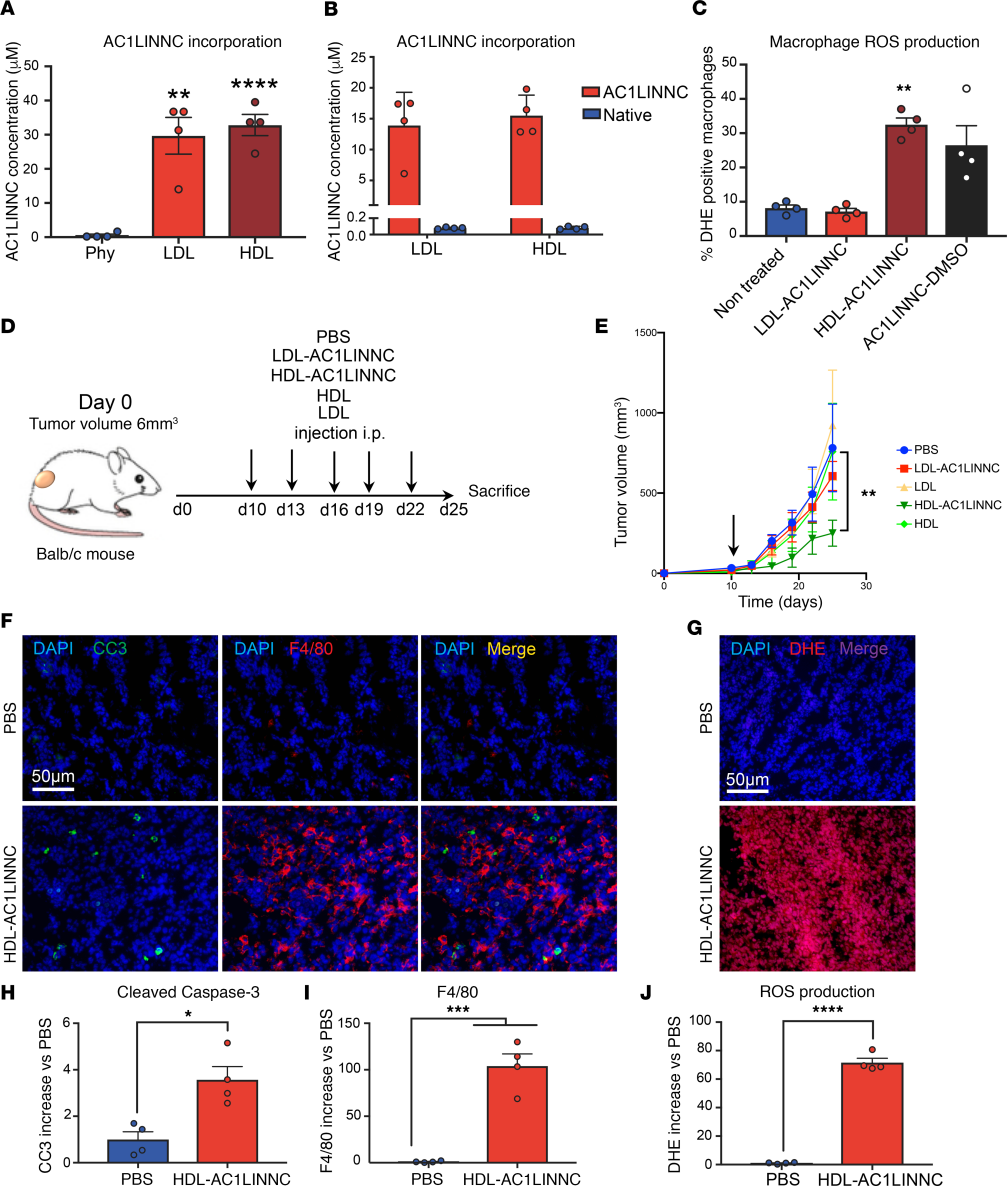
HSP70 inhibitor vectorization in HDL prevents tumor growth by targeting macrophages. (**A**) LDL and HDL were purified by density gradient ultracentrifugation. Total cholesterol in lipoproteins was adjusted to 1 mM. Lipoproteins were then incubated with AC1LINNC (100 μM) for 3 hours at 37°C and were then submitted to dialysis. Total AC1LINNC concentration in lipoproteins was then measured by Mass spectrometry. (**B**) For AC1LINNC exchanges, after integration, AC1LINNC-bound LDL or AC1LINNC-bound HDL (0.5 μM) were incubated for 24 hours at 37°C with native HDL or LDL (0.5 μM), respectively. LDL and HDL were purified by density gradient ultracentrifugation, and total cisplatin concentration in lipoproteins was then measured. Data are represented as mean value ± SEM. *n* = 4, **P < 0.01, ***P < 0.001. (**C**) For macrophages activation, human M2 macrophages were treated for 2 hours with AC1LINNC (10 μM in DMSO) or vectorized in LDL or HDL (10 μM final AC1LINNC concentration). Percentage of ROS^+^ macrophages are represented as mean value ± SEM. *n* = 4, **P < 0.01. (**D**) Balb/c mice were injected with CT-26 colorectal cancer cells (1 × 10^6^ cells/mice, s.c.). At the indicated times, mice were treated either with HDL-PBS (100 μM cholesterol), LDL-AC1LINNC or HDL-AC1LINNC (100 μM cholesterol, 10 μM AC1LINNC, 100 μL/mouse), or native LDL/HDL. *n* = 4. (**E**) Tumor volume was measured every 3 days and represented as mean value ± SEM. ***P < 0.001; arrow indicates first injection. (**F**) Apoptosis and macrophage infiltration were determined in histological slides labeled with a cleaved caspase–3 antibody (green) and a F4/80 antibody (red), and with DAPI. Pictures were chosen in random fields and are representative of 5 pictures taken for each condition. *n* = 4. Scale bar: 50 μm. (**G**) ROS production in tumors was measured in histological slides by DAPI/DHE staining. Images, taken in random chosen fields, are representative of 5 pictures taken for each condition. *n* = 4. Scale bar: 50 μm. (**H**–**J**) Quantifications of the immunofluorescence intensity of cleaved caspase–3 (**H**), F4/80 (**I**), and DHE (**J**). Data are represented as mean increase versus PBS ± SEM. *n* = 4, *P < 0.05, ***P < 0.001, ****P < 0.0001. *P* values were calculated using 1-way ANOVA (**A** and **E**) or 2-tailed unpaired *t* tests (**H**–**J**).

With the rationale that HDLs abundantly accumulated in macrophages, we decided to complex HDL to a molecule that we screened for its ability to target the HSP70, a chaperone known for its tumorigenic role involving macrophages differentiation/activity(4, 9–11). This molecule, designated as AC1LINNC (Supplemental Figure 2A), binds to HSP70 with an IC_50_ of 0.2 μM (Supplemental Figure 2C) and inhibits HSP70 chaperone activity (Supplemental Figure 2, B and D). AC1LINNC is highly hydrophobic and insoluble, hampering its use in vivo and thereby increasing the interest for its vectorization. We incubated HDL or LDL (1 mM cholesterol each) with AC1LINNC (to a final concentration of 100 μM) (Supplemental Figure 3A). Mass spectrometry analysis revealed a 30% uptake of AC1LINNC by both LDL and HDL, achieving a final concentration of about 30 μM (Figure 4A). To evaluate the stability of AC1LINNC-bound lipoproteins and possible exchange of AC1LINNC between LDL and HDL particles, AC1LINNC-bound LDLs or AC1LINNC-bound HDLs (1 mg/mL) were incubated with native HDLs or LDLs (1 mg/mL), respectively (Supplemental Figure 3B). AC1LINNC was not detected in the newly added native lipoproteins, indicating that the interaction is stable with no exchange with other lipoproteins (Figure 4B). We found that, while the AC1LINNC complexed to LDL had no effect, the same amount of the AC1LINNC vectorized in HDL or solubilized in DMSO induced ROS production in macrophages (Figure 4C).

To confirm the in vivo potential interest of HDL-bound AC1LINNC, we used the rodent CT-26 colorectal cancer model. CT-26 cells were s.c. injected into the left flank of Balb/c mice. Day 0 was considered when tumor size reached about 6 mm^3^. At day 10, mice were treated i.p. with PBS, HDL-AC1LINNC (1.5 mg/kg), LDL-AC1LINNC, or native HDL or LDL. Injections were performed every 3 days until the end of the experiment (day 25, for ethical considerations; Figure 4, D and E). Consistent with data obtained from cultured macrophages, we observed higher macrophage tumor infiltration with a drastic increase in ROS production, along with a mild induction of apoptosis in cancer cells (Figure 4, F–J). That was associated with tumor regression (Figure 4E), thereby bringing proof of concept of a macrophage-guided effect of HDL complexes. Interestingly, we did not observe any effect of LDL-AC1LINNC treatment or of either native HDL or LDL on tumor growth.

### Lipoproteins as nanovectors for the chemotherapeutic drug cisplatin.

**Figure 5:**
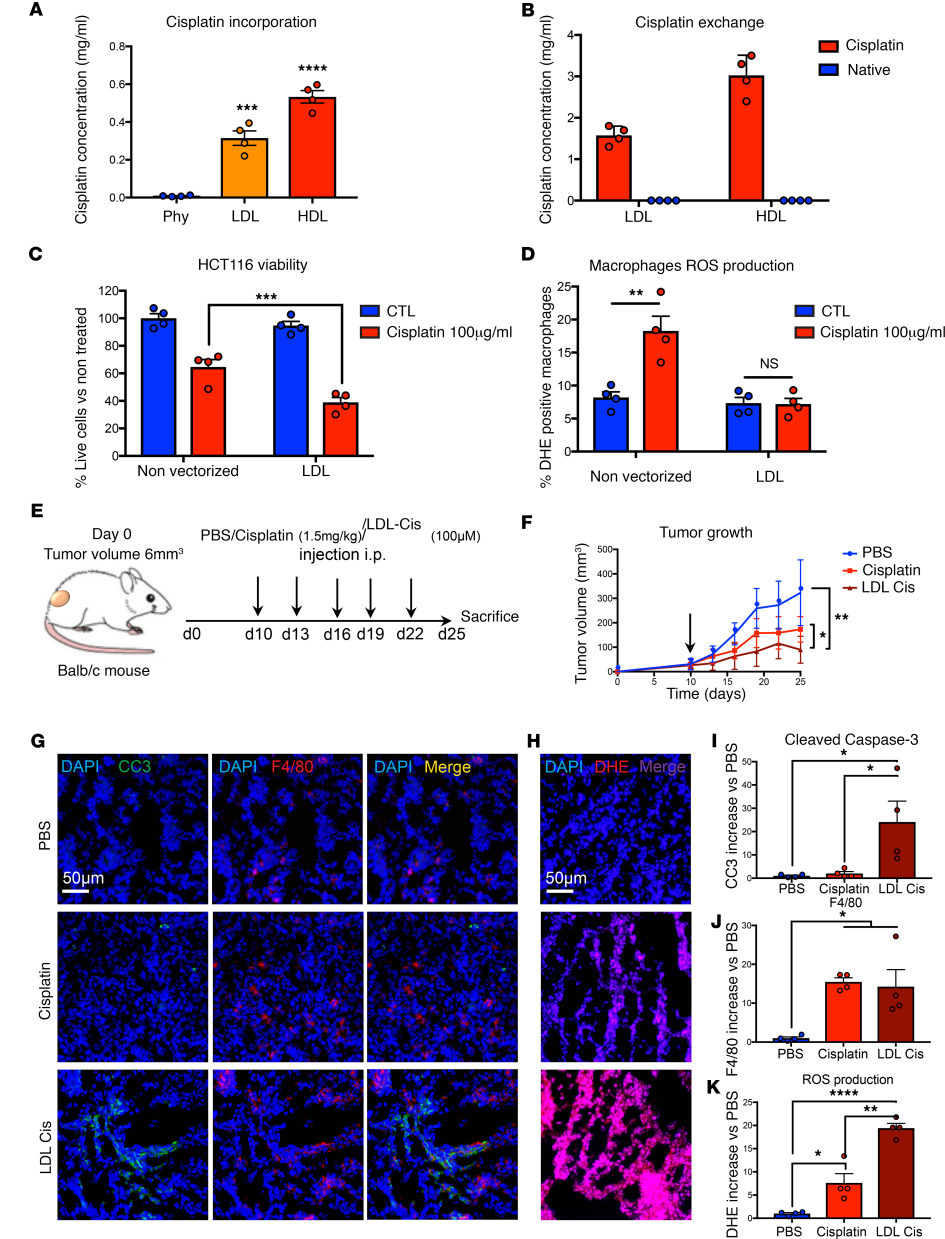
In vivo effects of cisplatin vectorization in LDL. (**A**) LDL and HDL were purified by density gradient ultracentrifugation. Total cholesterol in lipoproteins was adjusted to 1 mM. Lipoproteins were then incubated with cisplatin (10 mg/mL) for 3 hours at 37°C and then submitted to dialysis. Total cisplatin concentration in lipoproteins was then measured by GF-AAS. (**B**) For cisplatin exchanges, after integration, cisplatin-bound LDL or cisplatin-bound HDL (0.5 μM) were incubated for 24 hours at 37°C with native HDL or LDL (0.5 μM), respectively. LDL and HDL were purified by density gradient ultracentrifugation, and total cisplatin concentration in lipoproteins was then measured. Data are represented as mean value ± SEM. *n* = 4, ***P < 0.001, ***P < 0.0001. (**C**) For the antitumor effect, HCT116 cells were treated for 48 hours with cisplatin alone or vectorized in LDL (25 μM final cisplatin concentration). Cell number is represented as mean percentage value versus nontreated ± SEM. *n* = 4, ***P < 0.001 versus nontreated. (**D**) For macrophages activation, human M2 macrophages were treated for 2 hours with cisplatin alone or vectorized in LDL (25 μM final cisplatin concentration). Percentages of ROS^+^ macrophages are represented as mean values ± SEM. *n* = 4, **P < 0.01. (**E**) Balb/c mice were injected with CT-26 colorectal cancer cells (1 × 10^6^ cells/mice, s.c.). At the indicated times, mice were treated either with LDL-PBS (100 μM cholesterol), cisplatin (1.5 mg/kg), or LDL-Cis (100 μM cholesterol, 1.5 mg/kg cisplatin). Five mice/group (*n* = 3). (**F**) Tumor volume was measured every 3 days and represented as mean value ± SEM. *P < 0.05, **P < 0.01; arrow indicates first injection. (**G**) Apoptosis and macrophage infiltration were determined in histological slides labeled with a cleaved caspase-3 antibody (green) and a F4/80 antibody (red), and with DAPI. Images were chosen in random fields and are representative of 5 images taken for each condition. *n* = 5. Scale bar: 50 μm. (**H**) ROS production in tumors was measured in histological slides by DAPI/DHE staining. Images, taken in random fields, are representative of 5 different ones taken for each condition. *n* = 5. Scale bar: 50 μm. (**I**–**K**) Quantifications of the immunofluorescence intensity of cleaved caspase-3 (**I**), F4/80 (**J**), and DHE (**K**). Data are represented as mean increase versus PBS ± SEM. *n* = 4. *P < 0.05, **P < 0.01, ****P < 0.0001. *P* values were calculated using 1-way ANOVA (**A**, **F**, and **I**–**K**) or 2-tailed unpaired *t* tests (**C** and **D**).

We next used cisplatin, an efficient drug whose heavy undesirable effects hamper its use. Lipoproteins were incubated with cisplatin (final concentration of 1 mg/mL) for 4 hours at 37°C (Supplemental Figure 3A). After dialysis, total cisplatin concentration in lipoproteins was measured by graphite furnace coupled to atomic absorption spectrophotometry (GF-AAS). We observed an integration of 30% and 50% of the initial dose of cisplatin, into LDL and HDL subfractions, respectively (Figure 5A). As previously described for the AC1LINNC (Supplemental Figure 3B), no cisplatin exchange with other lipoproteins was detected (Figure 5B). In silico (Supplemental Figure 4, A and B, and Supplemental Table 4) and raman spectroscopy studies (See Supplemental Figure 4C) indicated that cisplatin bound cysteine residues of the ApoB-100 protein.

### Antitumor in vivo effect of cisplatin complexed to LDL.

The impact of cisplatin-LDL versus -HDL complexes on in vitro tumor cell death was determined by 3-(4,5-dimethylthiazol-2-yl)-2,5-diphenyltetrazolium bromide (MTT) viability test. We observed that LDL vectorization improved cisplatin-induced cancer cell mortality (Figure 5C), while HDL vectorization had no effect compared with the control (Supplemental Figure 5A). These results were in agreement with our previous data showing that HDLs were not captured by HCT116 tumor cells, while LDLs hardly entered macrophages. Consistently, we observed that cisplatin complexed to LDL had no effect on macrophage ROS production (Figure 5D), while HDL-bound cisplatin induced a strong response (Supplemental Figure 5B).

This effect of the LDL-cisplatin (LDL-Cis) complexes in tumor cells was further observed in vivo in CT-26 tumor–bearing mice (Figure 5, E–K). Mice were treated i.p. every 3 days with PBS, native HDL or LDL, cisplatin (Cis-Pt, 1.5 mg/kg), or cisplatin (1.5 mg/kg) complexed to LDL (LDL-Cis) or HDL (HDL-Cis) (Figure 5E). LDL-Cis treatment was associated with a stronger tumor regression than cisplatin alone (Figure 5F), while no significant effects of HDL-Cis or native lipoproteins were observed on tumor growth (Supplemental Figure 5C). Consistently, we observed that LDL vectorization of cisplatin was associated with a higher tumor cell death (cleaved caspase-3; Figure 5, G and I) and a higher proportion of macrophages infiltrating the tumor (F4/80; Figure 5, G and J), compared with both control and nonvectorized cisplatin groups. Interestingly, cleaved caspase-3 staining did not colocalize with F4/80, suggesting a selective proapoptotic effect on tumor cells. Additionally, this proapoptotic effect was associated with increased ROS production, as assessed by DHE/DAPI staining (Figure 5, H and K). To summarize, LDL vectorization improves the efficiency of cisplatin by increasing tumor cell death and, most probably indirectly, favoring cytotoxic macrophage infiltration.

### Vectorization of cisplatin by LDL strongly diminishes cisplatin adverse side effects.

**Figure 6:**
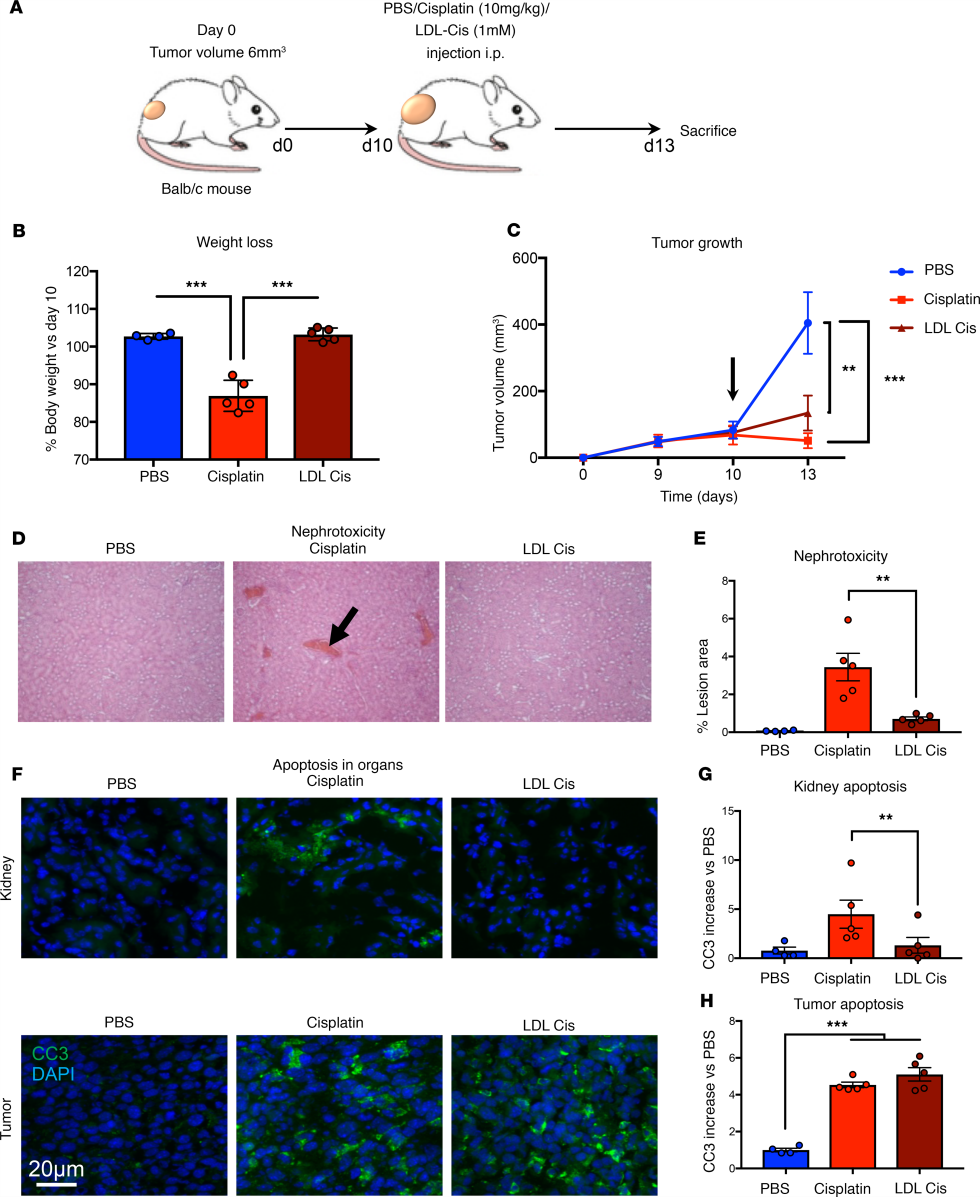
LDL vectorization of cisplatin diminishes cisplatin adverse effects. (**A**) Balb/c mice were injected with CT-26 colorectal cancer cells (1 × 10^6^ cells/mice, s.c.). When tumors reached approximately 300 mm^3^ (by day 10), mice were i.p. injected with either LDL-PBS (100 μM cholesterol, *n* = 4), cisplatin (20 mg/kg, *n* = 5), or LDL-Cis (100 μM cholesterol, 20 mg/kg cisplatin, *n* = 5). (**B** and **C**) Mice were euthanized 4 days later, and weight loss (**B**) and tumor volume (**C**) were determined. Data are represented as mean value ± SEM. **P < 0.01, ***P < 0.001; arrow indicates first injection. (**D**) To evaluate cisplatin nephrotoxicity, kidney samples were recovered for IHC H&E staining. Total magnification was 10 × 20. Images, taken in random fields, are representative of 5 pictures taken for each condition. *n* = 4–5 mice per group. (**E**) Quantification of the percentage of lesion area reported to total field area (black arrow) is represented as mean value ± SEM. **P < 0.01. (**F**) Apoptosis in organs was assessed by immunofluorescence in kidney and liver. Histological slides were labeled with a cleaved caspase-3 antibody (green) and with DAPI. Images are representative of 5 pictures taken for each condition. *n* = 4. Scale bar: 20 μm. (**G** and **H**) Quantifications of the immunofluorescence intensity of cleaved caspase-3 in kidneys (**G**) or tumor (**H**) sections. Data are represented as mean increase versus PBS ± SEM. *n* = 4–5 mice per group. **P < 0.01, ***P < 0.001. *P* values were calculated using 1-way ANOVA.

To investigate the impact of lipoprotein vectorization on cisplatin nephrotoxicity, mice were treated with cisplatin using a standardized kidney injury protocol. Tumor-bearing mice were injected with a single dose of 30 mg/kg cisplatin and euthanized at day 3. Toxicity was assessed by evaluating weight loss and renal dysfunction (Figure 6A). Mice treated with Cis-Pt experienced a 19% weight loss only 3 days following injection. In contrast, mice treated with LDL-bound cisplatin did not experience any weight loss (Figure 6B), while, interestingly, cisplatin anticancer drug efficiency was still observed (Figure 6C).

Following cisplatin treatment, mice displayed distortion of the overall renal morphology, dilation of renal tubules, and appearance of protein cast, most of which were significantly attenuated in the cisplatin lipoprotein-vectorized group (Figure 6, D and E). In addition to these observations, apoptosis assessed by caspase-3 cleavage was dramatically decreased in the kidney of mice treated with cisplatin complexed to LDL (Figure 6, F and G), while the induction of apoptosis in tumor cells was comparable in both groups (Figure 6, F and H). Furthermore, since the liver is involved in lipoprotein turnover, we aimed to assess if cisplatin complexed to LDL would induce hepatic toxicity. Interestingly, we found that LDL-bound cisplatin did not induce hepatic toxicity, as shown by comparing apoptosis (cleaved caspase-3) in the liver of animals treated with cisplatin versus LDL-cisplatin (Supplemental Figure 1, C and D). Altogether, cisplatin complexed to LDL, while preserving cisplatin antitumor effect, displayed a reduced side effect toxicity.

### Combinational effect of AC1LINNC-HDL and cisplatin-LDL complexes.

**Figure 7:**
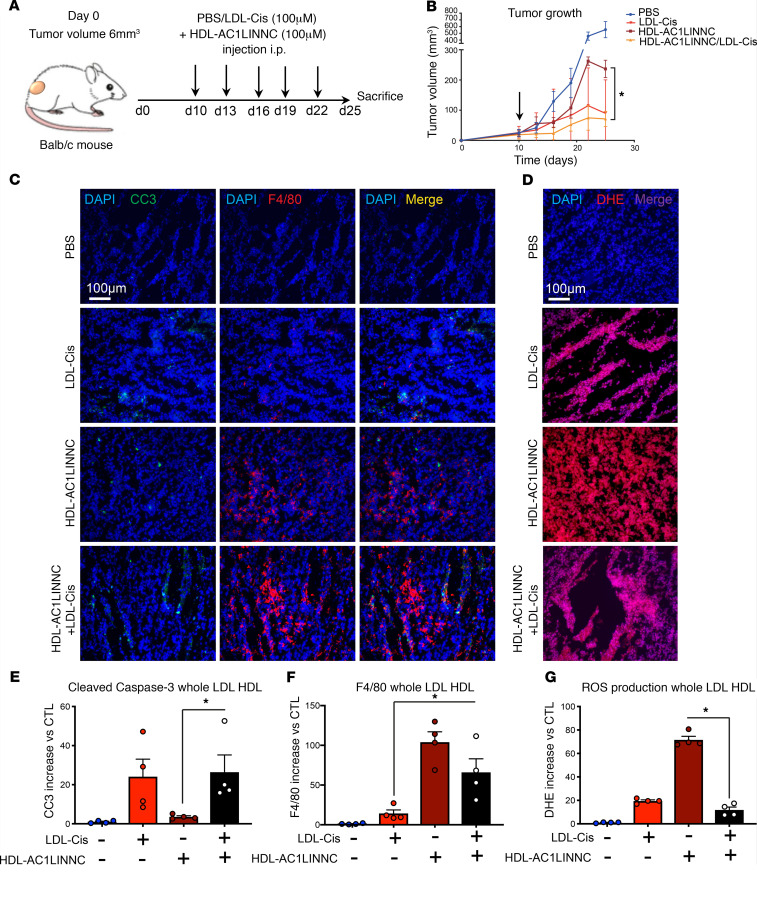
In vivo additive effect of cisplatin-loaded LDL and AC1LINNC-loaded HDL. (**A**) Balb/c mice were injected with CT-26 colorectal cancer cells (1 × 10^6^ cells/mice, s.c.). At the indicated times, mice were treated either with PBS, HDL-AC1LINNC (100 μM cholesterol, 10 μM AC1LINNC, 100 μL/mouse), or LDL-Cis (100 μM cholesterol, 1.5 mg/kg cisplatin) + HDL-AC1LINNC (100 μM cholesterol, 10 μM AC1LINNC, 100 μL/mouse). *n* = 5 mice per group. (**B**) Tumor volume was measured every 3 days and represented as mean value ± SEM. *P <0.05; arrow indicates first injection. (**C**) Apoptosis and macrophage infiltration were determined in histological slides labeled with a cleaved caspase-3 antibody (green) and a F4/80 antibody (red), and with DAPI. Images were chosen in random fields and are representative of 5 pictures taken for each condition. *n* = 5. Scale bar: 50 μm. (**D**) ROS production in tumors was measured in histological slides by DAPI/DHE staining. Pictures, taken in random fields, are representative of 5 pictures taken for each condition. *n* = 5. Scale bar: 50 μm. (**E**–**G**) Quantifications of the immunofluorescence intensity of cleaved caspase-3 (**E**), F4/80 (**F**), and DHE (**G**). Data are represented as mean increase versus nontreated ± SEM. *n* = 4, *P < 0.05, ****P < 0.0001. *P* values were calculated using 1-way ANOVA.

Finally, we tested the impact of the association of LDL-Cis complexes together with AC1LINNC-HDL complexes. Tumor-bearing mice were treated with LDL-Cis, HDL-AC1LINNC, or the combination of both. While we did not observed a stronger decrease in tumor growth when using the combinational therapy (Figure 7, A and B), immunofluorescent staining revealed (a) a strong induction of cancer cell apoptosis (Figure 7, C and E), comparable with that observed in the animals treated with LDL-Cisplatin alone and (b) a strong burst in macrophage infiltration (Figure 7, C and F) comparable with HDL-AC1LINNC alone. Overall ROS production was reduced compared with HDL-AC1LINNC alone but comparable to LDL-Cis alone (Figure 7, D and G). This suggests that this combined strategy aiming to simultaneously target cancer cells with one drug (cisplatin-LDL) and tumor-infiltrating macrophages with the other (AC1LINNC-HDL) allows a complementary additive effect that could prove more efficient during prolonged treatment.

## Discussion

In the field of cancer treatment, liposomal encapsulation of lipophilic drugs such as doxorubicin (Doxil, AmBisome) or vincristine (Marquibo) (12, 13) led to improved efficacy and safety. These new formulations were approved by the US Food and Drug Administration (FDA) (12, 13). However, the use of liposomal carriers for hydrophilic cisplatin has not achieved these goals so far. Among the few liposomal formulations of cisplatin that reached clinical trials, encapsulation of cisplatin in SPI-77 liposomes did not produce significant clinical efficacy in phase II studies despite its safer toxicity profile (14), due to inefficient release of the drug from the carrier (15). Although Lipoplatin (active encapsulation of cisplatin into PEGylated liposomes) showed enhanced antitumor efficacy compared with free cisplatin in some patients, along with reduced renal toxicity, phase II and III studies demonstrated inconsistent effects on survival rates (16). Finally, LiPlaCis, a phospholipase A2–sensitive liposomal cisplatin carrier, has shown a poor safety profile, leading to the discontinuation of phase I studies (17).

In the present study, we considered native human LDL and HDL as carriers for several reasons: (a) lipoproteins are endogenous, physiological and stable molecular complexes that can carry a wide variety of molecules in the bloodstream; (b) they bear specific apolipoproteins that allow targeting to specific receptors; and (c) certain chemotherapeutic drugs such as cisplatin display affinity for plasma proteins and therefore are likely to bind to apolipoproteins (18). This latter hypothesis was supported in the present work by in silico studies, showing that hydrophobic cisplatin interacts with the protein moiety and not the lipid moiety of lipoproteins. Furthermore, we demonstrate in vitro that cisplatin association to LDL and HDL is highly efficient and stable in aqueous solution, with no subsequent release of bound cisplatin into the buffer or toward other nonloaded lipoproteins. These results, together with our in vivo kinetics experiments, suggest that cisplatin binding to lipoproteins can significantly prolong its half-life in the blood (only 0.24 hours for free cisplatin; ref. 19). This may allow the molecule to reach its target tissue instead of being quickly cleared in an unspecific manner. Accordingly, we were able to show that substantial amounts of labeled lipoproteins could be found within CT-26 tumors after their systemic administration in mice.

Interestingly, LDL and HDL targeted different cells within the tumor. While labeled HDL preferentially accumulated in tumor macrophages, LDL were mainly found within CT-26 cancer cells. A possible explanation is that LDL receptor (LDL-R) expression is abnormally elevated in cancer cells (20, 21). In contrast, HDL, which uptake cholesterol excess from peripheral cells back to the liver, have been shown to interact with immune cells via specific membrane transporters and scavenger receptors, particularly present in macrophages. The specific tropism of the LDL for tumor cells may explain the lack of cisplatin’s side effects, such as renal and hepatic toxicity, when conjugated to LDL.

Since HDL particles are abundantly taken up by macrophages, we believe that loading of HDL with immunotherapeutic compounds may be more promising than with chemotherapeutic drugs. Thus, HDL might have a strong therapeutic interest to vectorize molecules that influence macrophage polarization toward an antitumor phenotype, and proof of the concept is shown in this work with an HSP70 inhibitor affecting macrophages differentiation and activation (9, 10). Overall, we conclude that the usage of HDL might allow in the future a more targeted and safer administration into the patient of immunotherapeutic agents alone or in combination with LDL-vectorized chemotherapeutic agents, such as cisplatin.

## Methods

Supplemental Methods are available online with this article.

### Cell culture and mice.

HCT116 cells (CCL-247) were from the American Type Culture Collection (ATCC). Human macrophages were isolated from healthy donor buffy coats (EFS Besançon) and differentiated as described (22). Six- to 8-week-old female Balb/c mice were from Charles River Laboratories. Mouse colon carcinoma cells CT-26 (CRL-2638 ATCC) were injected s.c. in the left flank. Time 0 was considered when tumor size reached 6 mm^3^. Mice were treated with cisplatin (1.5 mg/kg, MilliporeSigma, 479306-1G) or LDL-Cis (100 μM cholesterol, 1.5 mg/kg cisplatin). For toxicity experiments, mice were injected i.p. at day 10 with PBS, cisplatin (20 mg/kg), or LDL-Cis (100 μM cholesterol, 20 mg/kg cisplatin). Tumors were measured every 3 days.

### Lipoprotein purification.

LDL and HDL were purified from plasma (EFS Besançon) as described (23). For biodistribution experiments, lipoproteins were labeled with DOTA-Bodipy (24), a fluorescent probe (excitation/emission spectra at 522/529 nm maxima) combined to a radioactive trap. Briefly, LDL or HDL (2 mg of protein) were incubated 4 hours at 37°C in bicarbonate buffer containing 565 μg DOTA-Bodipy (synthesized in-house) (25). Bicarbonate buffer and unbound labels were removed, and lipoproteins were recovered in sterile PBS by filtration in centrifugal concentration tubes (30 kDa cutoff Centricon, MilliporeSigma).

### Cisplatin and AC1LINNC incorporation in lipoproteins.

Cisplatin (MilliporeSigma, 479306-1G) was dissolved in 0.9% NaCl to a 10 mg/mL concentration. AC1LINNC was diluted DMSO to a 100 μM concentration. Cisplatin or AC1LINNC solutions were diluted 10 times in 1 mM LDL or HDL fractions and incubated 4 hours at 37°C. Unbound cisplatin, AC1LINNC, and DMSO were removed by successive dialyses using Spectrum Spectra/Por 1 RC Dialysis Membrane Tubing (6000–8000 Da cutoff, Thermo Fisher Scientific, 08-670C). For LDL/HDL-Cis exchange assays, cisplatin-bound LDL or cisplatin-bound HDL (1 μM) were incubated 24 hours at 37°C with native HDL or LDL (1 μM). Cisplatin incorporation in LDL and HDL was then assessed by GF-AAS and AC1LINNC incorporation by mass spectrometry.

### Biodistribution and pharmacokinetics imaging.

Tumor-bearing Balb/c mice (~300 mm^3^) received 5 μg ^111^In-DOTAGA-HDL or ^111^In-DOTAGA-LDL (8–10 MBq); i.v. SPECT/CT dual imaging was performed 1 hour, 24 hours, and 72 hours after injections using a NanoSPECT/CT small animal imaging tomographic γ-camera (Bioscan Inc.). Mice were anesthetized with isoflurane (1.5%−3% in air) and positioned in a cradle. CT (55 kVp, 34 mAs) and helical SPECT acquisitions were performed in immediate sequence. Both ^111^In photo peaks (171 and 245 keV) were used with 10% wide energy windows. Radioactivity was measured (from blood, tumor, and organs) with a scintillation γ-counter. Data were then converted to percentage of injected dose per gram of tissue (%ID/g). SPECT/CT fusion image was obtained using the InVivoScope software (Bioscan Inc.). Radioactivity contents from image analysis were expressed in Bq/g, converted to percentage of injected dose, and compared with those determined by ex vivo counting.

### Statistics.

Differences among 2 groups were determined using 2-tailed unpaired Fisher’s *t* test and 1-way ANOVA, followed by Bonferroni’s multiple comparison test for more than 2 groups, using SigmaStat version 3 (GraphPad Software). Differences were considered significant when *P* < 0.05.

### Study approval.

All animal procedures and experiments were approved by the ethical committee of the Université de Bourgogne (protocol N3613).

## Author contributions

TH performed in vitro and in vivo experiments and participated in manuscript redaction. CR, SY, and TM performed in silico calculations, SF ran raman experiments, and they all participated in manuscript redaction. TG provided lipoproteins. PSB analyzed radiolabeled images. TL performed in vitro experiments. AS performed pharmacokinetics analyses. RD performed radiolabeled lipoprotein injections. LD participated to in vivo experiments. LP provided lipoproteins. SM provided AC1LINNC. MW and FN dosed the cisplatin and provided discussion. MM and FD provided Bodipy. BC performed radiolabeled mice imaging. LL, CG, and FL designed the study, supervised the work, and wrote the manuscript.

## Supplementary Material

Supplemental data

## Version 1 11/30/2020

In-Press Preview

## Version 2 12/17/2020

Electronic publication
