# Supplementary material for: Lipoproteins LDL versus HDL as nanocarriers to target either cancer cells or macrophages
Source: JCI Insight. 2020 Dec 17;5(24):e140280. doi: 10.1172/jci.insight.140280 (PMC7819744; doi:10.1172/jci.insight.140280)
Supplement: Supplemental data [file jciinsight-5-140280-s121.pdf]

Supplementary material

Supplementary figures:

Supplementary figure 1.

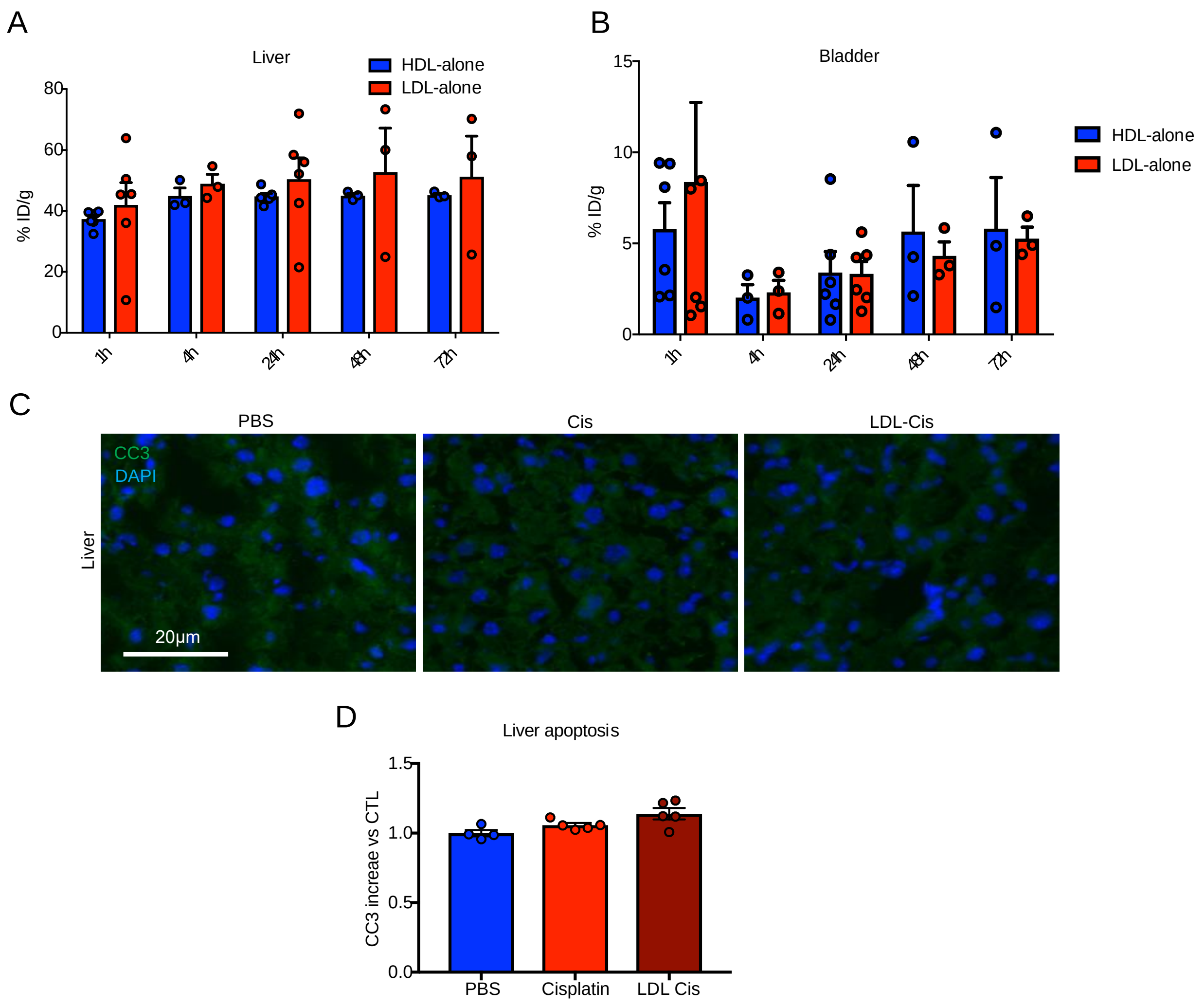

Supplemental Data 1: Kinetics of LDL/HDL incorporation in the liver and the bladder.

A, B: Female Balb/c mice (n=6) were grafted by subcutaneous injection of 1.10^6 colon tumor cells CT26. When tumor reached approximately 300 mm^3, tumor bearing-mice were given 5 µg ^{111}In-DOTAGA-HDL (blue histogram) or ^{111}In-DOTAGA-LDL (8−10MBq) (red histogram) by intravenous injection. SPECT/CT dual imaging was performed 1h, 24h and 72h after the injection of the radiolabeled conjugate using a

NanoSPECT/CT small animal imaging tomographic γ-camera. CT (55 kVp, 34 mAs) and helical SPECT acquisitions were performed in immediate sequence. Radioactivity was measured with a scintillation γ-counter from tumor and heart   Data were then converted to percentage of injected dose per gram of tissue (%ID/g). SPECT/CT fusion image was obtained using the InVivoScope software. Radioactivity in liver **(A)** and bladder **(B)** was measured was measured with a scintillation γ-counter. Data is presented as mean value +/- SEM. **C:** Hepatotoxicity: Balb-c mice were injected with CT-26 colorectal cancer cells (10^6 cells/mice, *s.c.*). When tumors reached approximately 300 mm^3 (by day 10), mice were i.p. injected with either PBS (n=4), cisplatin (20 mg/kg, n=5), or LDL-Cis (100μM cholesterol, 20mg/kg cisplatin, n=5) Apoptosis were t-determined in histological slides labeled with a cleaved caspase-3 antibody (Green), and with DAPI. Pictures were chosen in random fields and are representative of five pictures taken for each condition. n=4, scale bar = 20μm. **D:** Quantifications of the immuno-fluorescence intensity of Cleaved Caspase-3. Data are represented as mean increase vs. CTL +/- SEM. n=4, *: $p<0.05$.

Supplementary figure 2.

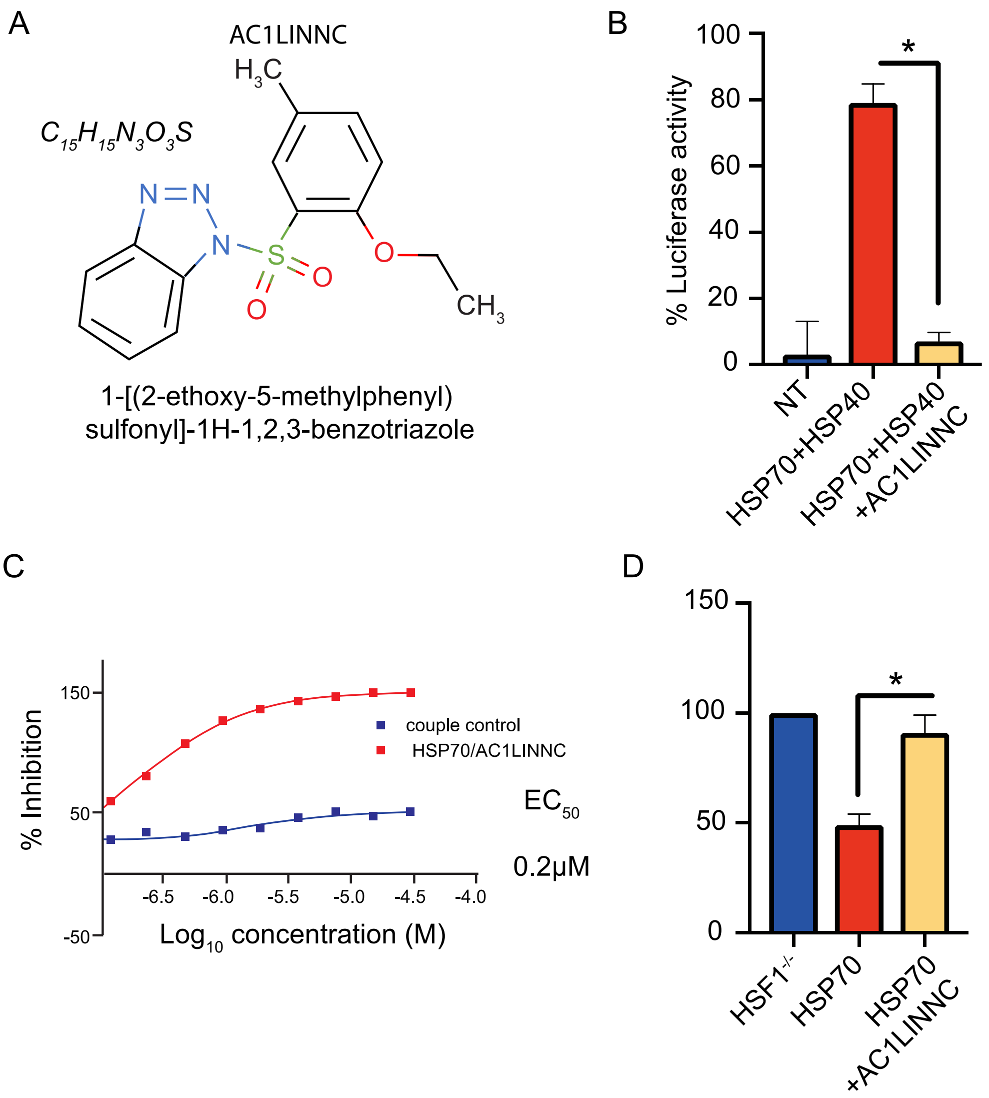

Supplemental Data 2: Characteristics and inhibitory properties of HSP70 inhibitor AC1LINNC

A: AC1LINNC empirical formula. **B:** Percentage inhibition of HSP70-induced luciferase refolding and regain of function. Data are represented as mean percentage vs. native luciferase +/- SEM. n=4, *: $p<0.05$. **C:** Determination of AC1LINNC EC50. **D:** Percentage of protein aggregation in HSF1-/- cell supernatant treated with HSP70 in the presence or absence of AC1LINNC. Data are represented as mean percentage vs. native luciferase +/- SEM. n=4, *: $p<0.05$. *P* values were calculated using one-way ANOVA.

Supplementary figure 3.

A

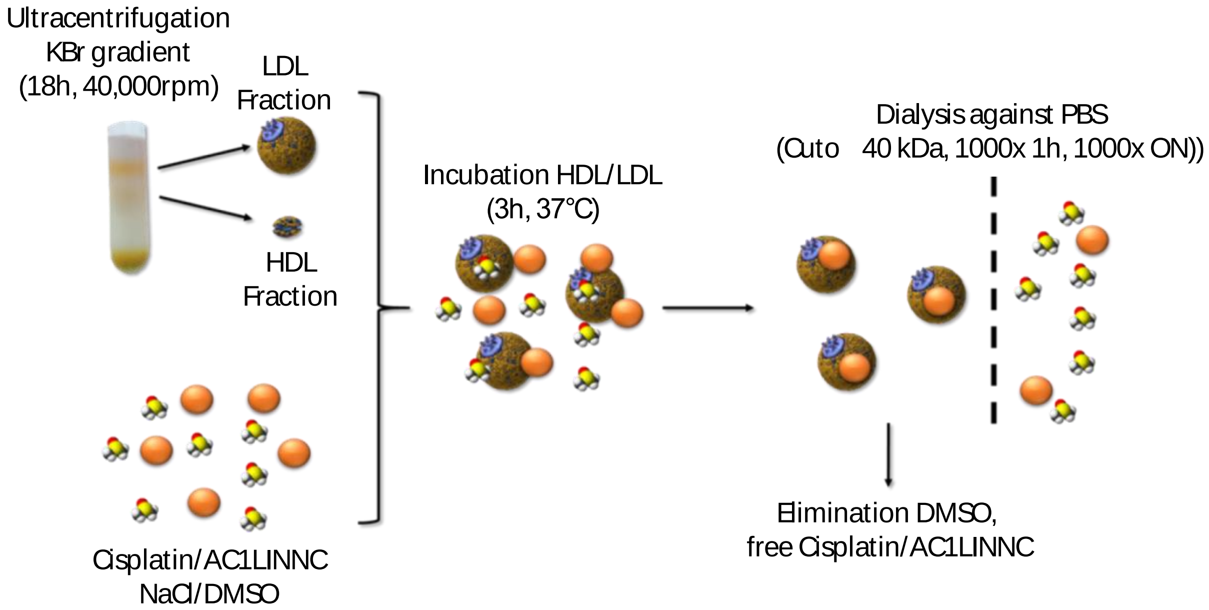

B

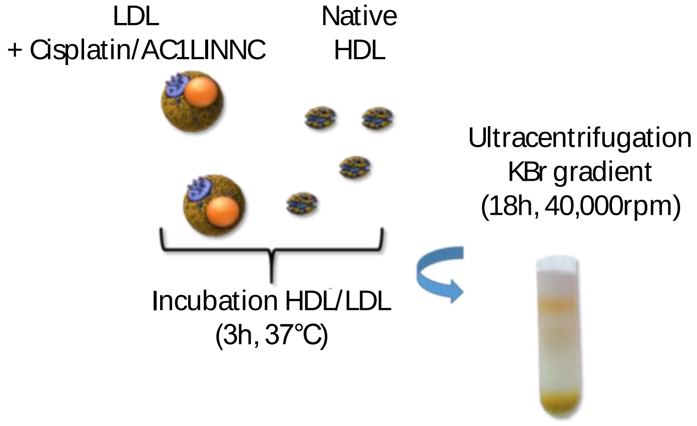

Supplemental Data 3: Schematic representation of LDL/HDL vectorization. A: Vectorization process.

B: exchange process.

Supplementary figure 4.

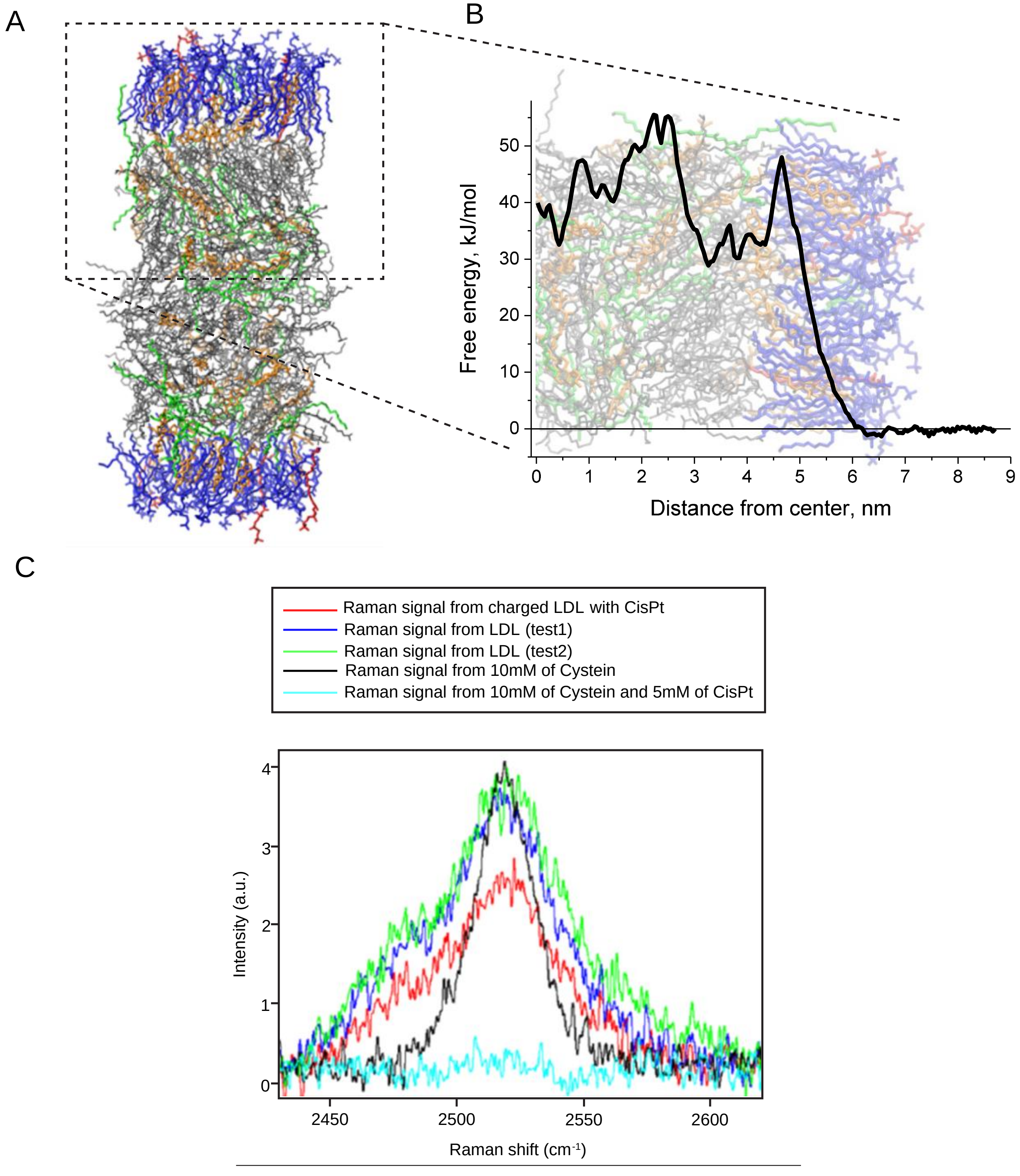

Supplemental Data 4: In silico analysis of Cisplatin integration in lipoprotein. A: Equilibrated model system used for potentials of mean force calculation. 1-palmitoyl-2-oleoyl-snglycero-3-phosphocholine (POPC) lipids are shown in blue, 1-palmitoyl-2-hydroxy-sn-glycero-3-phosphocholine (lyso PC) in red,

cholesterol in orange, cholesterol oleate in grey and glyceryl trioleate in violet. **B:** Potentials of mean force of transferring individual cisplatin molecule (black line) from bulk water to the core of model LDL lipid droplet. The plots are superimposed onto a Snapshot of the equilibrated model (dashed square) An arrow shows a position of minor energy minimum in the region of lipid head groups. **C**: Raman spectra of the SH stretching vibration LDL, LDL with cisplatin, 10 mM cysteine and 10 mM cysteine with 5 mM cisplatin.

Supplementary figure 5.

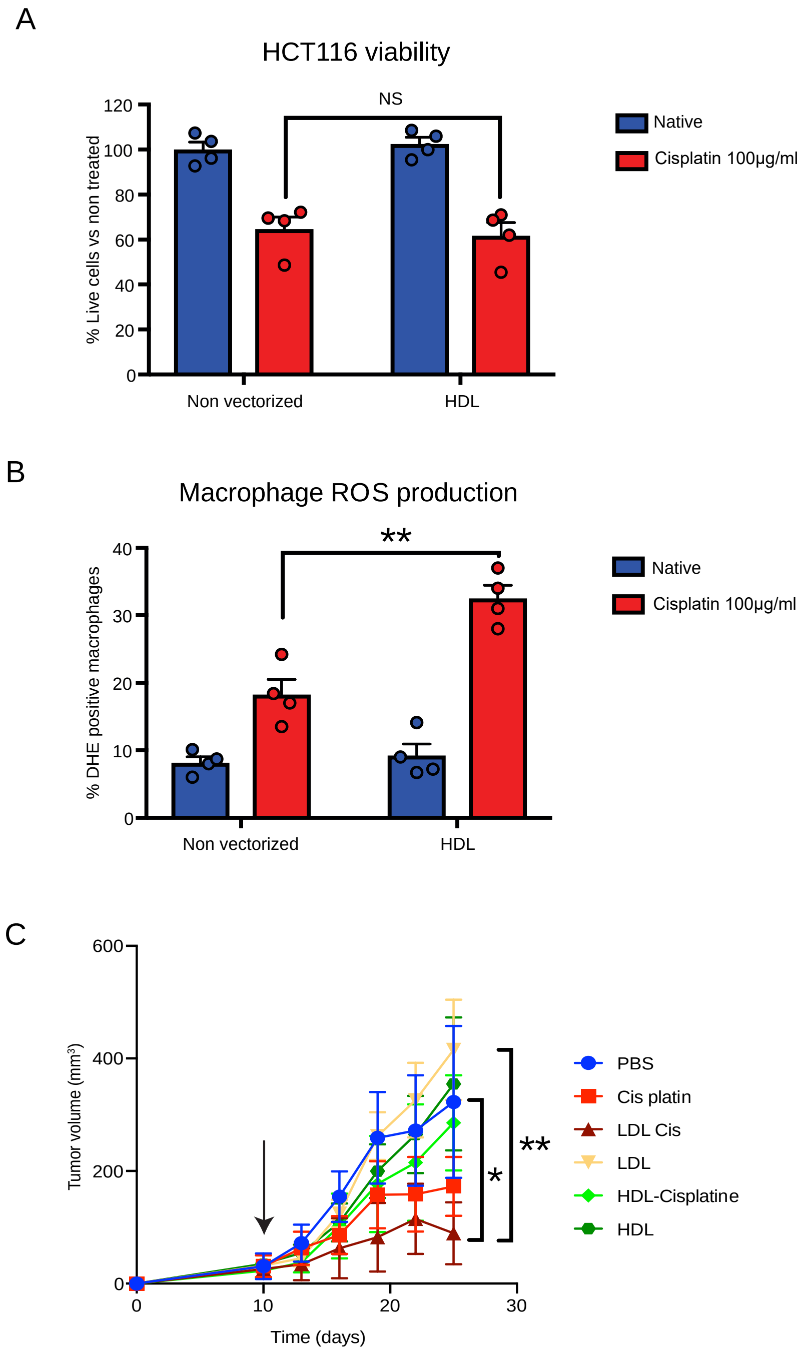

Supplemental Data 5: In vitro effects of cisplatin vectorization in HDL. A: For the anti-tumor effect, HCT116 cells were treated for 48 hours with cisplatin alone or vectorized in HDL (25µM final cisplatin concentration). Cell number is represented as mean percentage value vs. non-treated +/- SEM. n=4, ***:

p<0.001 vs. non-treated. #: p<0.05 vs. cisplatin alone**.** **B:** For macrophages activation, human M2 macrophages were treated for 2 hours with cisplatin alone or vectorized in HDL (25µM final cisplatin concentration). Percentage of ROS positive macrophages are represented as mean value +/- SEM. n=4, **: p<0.01, NS = Not Significant. **C:** Balb-c mice were injected with CT-26 colorectal cancer cells (10^6 cells/mice, *s.c.*). At the indicated times mice were treated either with PBS, cisplatin (1.5 mg/kg), LDL-Cisplatin or HDL-Cisplatin (100µM cholesterol, 1.5mg/kg cisplatin) or native LDL/HDL (100µM cholesterol). n=4. Tumor volume was measured every 3 days, and represented as mean value +/- SEM, *: p<0.05, **: p<0.01, arrow indicates first injection. *P* values were calculated using two-tailed unpaired t-tests **(A, B)** or one-way ANOVA **(C)**.

Supplementary Table 1.

Study	Unit	1	2	3	4	5
Dose	mg	0.0615	0.0615	0.0615	0.0615	0.0615
Cmax	mg/ml	0.0734476	0.11222	0.0809667	0.0738703	0.107501
Tmax	min	5	5	5	5	5
AUClast	mg/ml*min	42.5264	53.5996	42.9365	43.9433	45.933
thalf	min	927.639	845.629	812.849	995.584	730.367
Clearance	mL/min	0.00100099	0.0008361	0.00105768	0.00094842	0.00102731
Vss	mL	1.21426	0.896636	1.1135	1.21538	0.970976

Supplementary table 1: HDL individual PK parameters (Cmax, Tmax, AUC0–6 h, VSS/F, Cl/F, t1/2) determined using a standard non-compartmental approach (NCA). All of the NCA calculations were performed with Kinetica Software (Thermo Scientific, Philadelphia, PA).

Supplementary Table 2.

Study	Unit	1	2	3	4	5
Dose	mg	0.0689	0.0689	0.0689	0.0689	0.0689
Cmax	mg/ml	0.0167727	0.0274689	0.0309725	#0.00899143	0.0417511
Tmax	min	5	5	5	#120	5
AUClast	mg/ml*min	5.00199	5.67125	5.23137	3.87094	3.85326
thalf	min	388.376	565.532	2288.37		
Clearance	mL/min	0.00790289	0.00621914	0.00205622		
Vss	mL	1.21426	0.896636	1.1135		0.970976

Supplementary table 2: LDL individual PK parameters (Cmax, Tmax, AUC0–6 h, VSS/F, Cl/F, t1/2) determined using a standard non-compartmental approach (NCA). All of the NCA calculations were performed with Kinetica Software (Thermo Scientific, Philadelphia, PA).

Supplementary Table 3.

	HDL		LDL	
	Estimation Mean	Interindividual variability (CV(%))	Estimation Mean	Interindividual variability (CV(%))
Clearance (mL/min)	0.0015	NA	1.10^{-6}	NA
Volume of central compartment (mL)	0.228	33.3%	2.29	32.3%
Intercompartmental clearance (mL/min)	0.144	NA	0.0366	NA
Volume of peripheral compartment (mL)	0.469	22.4%	6.33	NA
Additive residual error (mg/ml)	1.10^{-5}	NA	$2.87.10^{-6}$	NA

Supplementary table 3: Inter-individual variabilities associated to the population PK parameters.

Supplementary Table 4.

Molecule	Number
POPC	64
18:1 Lyso PC	8
Cholesterol	60
Cholesterol oleate	160
Glyceryl trioleate	18
Water	4900

Supplemental Table 4: Composition of the system used for production simulations. The number of lipid molecules correspond to 1/10th of the whole LDL particle used in figure 4.

Supplementary methods:
Cell survival experiments

50.000 cells were seeded in 96-well plates in 200µl RPMI-10% FBS and allowed to adhere Over Night at 37°C 5% CO_2. For cell stimulations, medium was removed and cells were stimulated for 48 hours with cisplatin alone or vectorized in LDL or HDL (25µM final cisplatin concentration) in OPTI-MEM medium. Following stimulations, cell survival was assessed by MTT assay[1].

In vivo Pharmacokinetics

Balb-c mice were injected *i.v.* with LDL-Bodipy and HDL-Bodipy at time 0, 100µl lipoprotein (1mM cholesterol). 100µl of blood was then dragged from the tail at t=5, 120, 300 & 480 minutes for LDL, and at t=5, 180, 300, 480 & 1440 minutes for HDL experiments. Blood samples were centrifuged 10 minutes at 1500 rpm and 25µl plasma was collected for lipoprotein extraction. Plasma density was adjusted to 1.21 by the addition of 1.42 density KBr solution (ddw, 0.1g/l EDTA, 0.02 g/l Sodium Azide), 350µl of 1.21 density KBr solution (ddw, 0.1g/l EDTA, 0.02 g/l Sodium Azide) were further added and samples were transferred in 450µl UltraClear centrifuge tubes (Beckman Coulter, 345843). Following centrifugation for 5h30 at 100,000 rpm, 20µl of floating lipoprotein was collected and bodipy fluorescence in the extracted lipoproteins was measured using a Victor5 spectrofluorimeter

PK analysis and modeling of LDL and HDL

HDL and LDL pharmacokinetics (PK) data were analyzed via a non-compartmental approach (Kinetica®, Thermo Scientific, Philadelphia, PA) to determine the major PK parameters (*i.e.*, Cmax, Tmax, AUClast, T½, clearance and Vss). Parameters were derived for each animal and then summarized as means or medians (for time). A second approach was applied in order to propose an optimal limited sampling strategy for the following studies. This approach, called population PK, is based on non-linear mixed effect

modeling (Monolix®, Lixoft, Paris). The aim is to model, *via* a compartmental approach, underlined by differential equations, the entire time course of the concentrations versus time.

Flow cytometry analysis

For macrophage ROS production analysis, cells were cultured for 30 min at 37 °C and 5%CO_2, in DHE (10 µM in PBS), scraped out and centrifuged (10 min, 1500 rpm, 4 °C). Cells were fixed for 5 min in a PBS 4%PFA solution and analyzed using an LSRII flow cytometer (Becton Dickinson). Primary Size-Granularity dot plot allowed us to discriminate cells from debris, and DHE positive cells were obtained by comparing red fluorescence *vs.* unstained samples.

For Bodipy integration, tumor-bearing Balb-c mice were injected *i.v.* with LDL-Bodipy and HDL-Bodipy at time 0, 100µl lipoprotein (1mM cholesterol). Tumors were recovered 12 hours after injections and processed for flow cytometry. Fresh CT-26 murine colorectal tumor samples were cut into small strips using a scalpel blade, transferred into a GentleMACS tube (Miltenyi Biotec, 130-093-237) and dissociated using the Multi Tissue Dissociation Kits (Miltenyi Biotec, 130-110-201, # 130-093-235). Dissociated tumor cells were fixed and stained with CD45-VioGreen® (,130-102-412 Miltenyi Biotechnologies), CD11b-APC (130-109-364, Miltenyi Biotechnologies), Ly6G-VioBlue® (130-102-227, Miltenyi Biotechnologies), F4/80-PE-Vio770 (130-102-193, Miltenyi Biotechnologies), (1/100 each in PBS-BSA 4%) and analyzed using an LSRII flow cytometer (Becton Dickinson). $CD45^-$ cells gated on a FSC-A/CD45 dot plot allowed to discriminate stromal cells from tumor leukocytes ($CD45^+$). Phagocytes within the leukocyte population were identified as $CD11b^+$ cells on a FSC-A/CD11b dot plot. Finally, macrophages were identified as $F4/80^+/Ly6G^-$ cells in the phagocyte population on a F4/80/Ly6G dot plot. The level of Bodipy integration in each population was measured in the FITC channel and gated on a SSC-A/FITC dot plot.

Immuno-histochemistry

Excised tumors were embedded in OCT (4585, Fisher Scientific), cut into 5-µm-thick sections, and fixed in cold PBS 2% paraformaldehyde solution (4 °C, 5 min). For toxicological analysis, Hematoxylin-Eosin (H&E) staining was performed by incubating the slides 1.5 min in eosin 515 LT (3801619, Leica Biosystems) and 2 min in hematoxylin 560 MX (3801575, Leica Biosystems).

For immuno-staining samples were incubated overnight at 4 °C with the primary anti-F4/80 antibody (BD Bioscience, 552958), and anti-Cleaved Caspase-3 (Cellsignaling, #9661) (1/200 dilution in blocking solution, PBS-2%BSA-0.1%TritonX100). Primary antibodies were detected using Alexa Fluor 488 or Alexa Fluor 647-coupled secondary antibodies (Invitrogen) (1/500 dilution). For ROS production, 10µM DHE solution was added to the fixed sections and incubated for 10 min at RT. Nuclear labeling was performed by incubating samples in DAPI (1 ng/ml in PBS, 30 s at RT). The slides were observed with a Nikon (E400, Eclipse) epifluorescence microscope. Five pictures were taken in random fields for each labeling, and analyzed using ImageJ software.

HSP70 chaperone activity.

HSP70 chaperone activity was evaluated with a protein thermolability assay as already described[1]. Cellular extracts, in which recombinant HSP70 were added with or without Molecule B (100µM), were diluted to a final concentration of 2 mg.mL-1 in pH 7 Tris-HCl buffer and heated at 55°C during 1 hour. After g×16,000 centrifugation at 4°C during 10 minutes, supernatant native protein quantity was determined by Lowry method (Dc Assay Kits, Bio-Rad). This final protein concentration was then compared to the initial protein concentration in supernatants to quantify denatured proteins.

For HSP70 refolding assay, Firefly luciferase (10 µM, Sigma-Aldrich) was denatured with 0.2 M guanidinium-HCl for 30 min at 25 °C[2]. Refolding of the denatured luciferase (80 nM) at 35 °C was monitored during 3 h in the presence of ATP (1 mM), Hsp40 (160 nM) and hHsp70 (800 nM) in the presence or absence of Molecule B (100µM).

Molecular dynamics simulations

Interaction of LDL with cisplatin was studied in atomic details by means of molecular dynamics simulations and quantum chemistry calculations. For the modeling of LDL lipid core we used a narrow column-like slice of the LDL particle which contained 10% of its whole volume with a lipid composition that corresponds to typical human LDL particle. The reader is referred to our previous work[3] for the details of system preparation and equilibration. All simulations were performed using Gromacs 5.1.2 package[4]. TIP3P water model was used. NPT conditions were used with a temperature of 320 K and a pressure of 1 bar maintained by v-rescale thermostat and Berendsen barostat respectively. The time step of 2 fs was used with all bonds converted to rigid constraints. Potential of mean force (PMFs) of transferring cisplatin molecule from water to the core of the LDL particle was computed by umbrella sampling simulations. The center of masses of the ligand was restrained by harmonic potential with the force constant of 2000 $kJ \cdot mol^{-1} \cdot nm^{-2}$ at different distances from the center of the slice. This resulted in 90 umbrella sampling windows with the step of 0.1 nm along the Z axis. Each window was simulated for at least 200 ns and the last 100 ns were used for analysis. PMFs were obtained with the weighted histogram technique (Kumar S. et al. Journal of Computational Chemistry, 1992. 13: 1011-21) as implemented in the Gromacs package.

Quantum chemistry calculations

The calculation of the thermodynamics and kinetics data for the different reactions of cisplatin with cysteine and methionine residues have been performed with Gaussian09 package (Gaussian 09, Revision E.01, M. J. Frisch et al 2009; Gaussian, Inc., Wallingford CT). All geometries (reactants, products and transition states) were optimized at the DFT/B3LYP level of theory. We used Pople-type basis sets : 6-31++G(d,p) on the C, H, O, N, Cl atoms and 6-311++G(d,p) on the sulfur atom. The platinum atom was treated with the Stuttgart-Dresden-Bonn quasirelativistic pseudopotential (SDD) for the core electrons, with two type f polarization functions[5]All the calculations were carried out in aqueous solution using the polarizable continuum model (PCM). The radii of atomic cavities were modified to 1.800 Å for sulfur and to 2.021 Å for platinum in order to obtain the best agreement between the calculated and experimental

solvation energies of sulfur-based ligands and cisplatin[6]. The Gibbs free energies of reaction were calculated as the difference between the Gibbs free energies of the two separate products and those of the two separate reactants. Energies barriers of reaction were calculated as the difference between the Gibbs free energy of the transition state and those of the two separate reactants.

Mass spectrometry

All solvents, LCMSMS quality grade, were purchased from Fischer Scientific (Illkirch, France). Twenty microliters of dialyzed lipoprotein fraction were mixed with 200 µl of acetonitrile for 5 min at room temperature. After centrifugation (15000g, +4°C, 10 minutes), clear supernatant was transferred to injection vials for prior LCMS² analysis. Targeted analysis of molecule B was realized on a 1200 6460-QqQ LC-MS/MS system equipped with an ESI-Jet stream source (Agilent technologies). Samples (5µL) were injected on a Zorbax Eclipse C8 2.1x100 mm, 1.8 µm column (Agilent technologies) at a flow rate of 0,2 ml/min, 50°C. Separation was achieved with a linear gradient of (solvent A) acetonitrile/methanol (95/5 v/v) and (solvent B) ammonium acetate 5 mmol/L in water as follows : 60% B for 4 min, down to 0% B in 11 min. Acquisition was performed in positive Single Reaction Monitoring (SRM) mode set-up with the following source parameters : temperature : 325°C, nebulizer gas flow rate was 10 L/min, sheath gas flow rate 11 L/min, temperature 350°C, capillary voltage 3500 V, nozzle voltage 1000 V). Transition used for detection of molecule B was 318.4→155 (fragmentor voltage 80 V, collision energy voltage 10 V). Area under curve of molecule B peak was determined with MassHunter Workstation Software version B.08. Finally, quantitation of molecule B was performed by calculating the ratio responses of each sample to a calibrated solution of molecule B in acetonitrile.

Vibrational spectroscopy of cisplatin/LDL interaction

The spectra reported were measured using aqueous solutions of either 10 mM of cysteine and 5 mM cisplatin (both from Sigma Aldrich) and LDL in phosphate buffer. The Raman spectroscopy experimental set up has previously been described[7]. The scattered light was detected at 90° using a

Princeton Instruments spectroscopy system which includes an Acton Spectra Pro 2500i monochromator with maximum resolution of 0.035 nm and a PIMAX-1024-RB CCD camera.

The laser intensity was constantly monitored by measuring the intensity of the N_2 Raman band as obtained from the laser beam scattering on air. All spectra were then corrected with respect to the changes in laser intensity by dividing them by the intensity of the N_2 Raman band acquisitioned on 400 laser shots. This is equivalent to a "normalization" of the measured intensities with respect to the Raman band of N_2 in air. Consequently, the intensities of the different experimental Raman spectra reported in the present paper are measured in the same (arbitrary) unit and can be directly compared.

Online only supplementary references

1. Gobbo J, Marcion G, Cordonnier M, *et al.* Restoring Anticancer Immune Response by Targeting Tumor-Derived Exosomes With a HSP70 Peptide Aptamer. J Natl Cancer Inst 2016;108(3).
2. Marcion G, Seigneuric R, Chavanne E, *et al.* C-terminal amino acids are essential for human heat shock protein 70 dimerization. Cell Stress Chaperones 2015;20(1):61-72.
3. Sobot D, Mura S, Yesylevskyy SO, *et al.* Conjugation of squalene to gemcitabine as unique approach exploiting endogenous lipoproteins for drug delivery. Nat Commun 2017;8:15678.
4. Wennberg CL, Murtola T, Pall S, *et al.* Direct-Space Corrections Enable Fast and Accurate Lorentz-Berthelot Combination Rule Lennard-Jones Lattice Summation. J Chem Theory Comput 2015;11(12):5737-46.
5. Martin JML, Sundermann A. Correlation consistent valence basis sets for use with the Stuttgart-Dresden-Bonn relativistic effective core potentials: The atoms Ga-Kr and In-Xe. Journal of Chemical Physics 2001;114(8):3408-3420.
6. Torrico F, Pappalardo RR, Marcos ES, *et al.* Hydration structure and dynamic properties of the square planar Pt(II) aquaion compared to the Pd(II) case. Theoretical Chemistry Accounts 2006;115(2-3):196-203.

7. Sjoberg B, Foley S, Cardey B, *et al.* An experimental and theoretical study of the amino acid side chain Raman bands in proteins. Spectrochim Acta A Mol Biomol Spectrosc 2014;128:300-11.
